# Identification and validation of reference genes for quantitative gene expression analysis under 409 and 415 nm antimicrobial blue light treatment

**DOI:** 10.3389/fmolb.2024.1467726

**Published:** 2025-01-06

**Authors:** Beata Kruszewska-Naczk, Mariusz Grinholc, Aleksandra Rapacka-Zdonczyk

**Affiliations:** Laboratory of Photobiology and Molecular Diagnostics, Intercollegiate Faculty of Biotechnology, University of Gdansk and Medical University of Gdansk, Gdańsk, Poland

**Keywords:** antimicrobial blue light, BestKeeper, *Escherichia coli*, geNorm, reference gene, RT-qPCR, NormFinder, RefFinder

## Abstract

**Introduction:**

Reverse transcription quantitative real-time polymerase chain reaction Q7 (RT‒qPCR) is a commonly used tool for gene expression quantification. Because the qPCR method depends on several variables that can influence the analysis process, stably expressed genes should be selected for relative gene expression studies. To date, there is insufficient information on the selection of appropriate reference genes for antimicrobial photodynamic inactivation (aPDI) and antimicrobial blue light (aBL) treatment. Therefore, the purpose of the present study was to determine the most stable reference gene under treatment with aBL under sublethal conditions and to evaluate differences in the expression of the selected gene after aBL treatment in comparison to the nontreated control.

**Methods:**

Selection of stable reference genes was performed using 4 programs: BestKeeper, geNorm, NormFinder and RefFinder under 409 and 415 nm aBL treatment.

**Results:**

The results revealed that the gene encoding the integration host factor β subunit (ihfB) in *Escherichia coli* was the most stably expressed gene after both 409 and 415 nm aBL treatment. Three programs, RefFinder, geNorm, and NormFinder, indicated that this gene had the most stable expression in comparison to the other reference gene candidates. The next best candidates were cysG, uidA, and gyrA. NormFinder revealed ihfB as the single gene and cysG - gyrA as the combination of reference genes with the best stability.

**Discussion:**

Universal reference genes are characterized by stable expression that remains consistent across various stress conditions. Consequently, it is essential to evaluate reference genes for each specific stress factor under investigation. In the case of aBL at different wavelengths, we identified genes that maintain stable expression following irradiation.

## Graphical Abstract

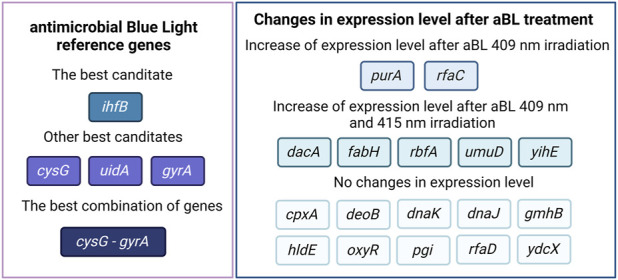

## Introduction

The increase in antibiotic resistance has been extensively reported (Hawkey, 2008; Robinson et al., 2016; Woc-Colburn and Francisco, 2020). The increasing resistance to antimicrobial agents and the lack of new molecules to combat bacterial resistance are serious problems and pose extremely dangerous health threats that modern medicine must address. The most important factor leading to multidrug resistance is the misuse and overuse of antibiotics (English and Gaur, 2010). Bacteria have continually evolved a repertoire of mechanisms to evade the effects of antibiotics (Enwemeka, 2013). Currently, approximately 700,000 people die every year due to infections caused by drug-resistant pathogens (O’Neill, 2016). To address this life-threatening public health problem, new antimicrobial strategies are being intensively sought. Among the most promising strategies are the application of antimicrobial photodynamic inactivation (aPDI) and antimicrobial blue light (aBL), which are the subjects of many basic and clinical studies. The main mechanism of action of aBL is based on reactive oxygen species generation under a specific light wavelength (in the range of 400–470 nm), which leads to bacterial eradication (Wang et al., 2017). To date, no development of bacterial resistance to aBL has been reported, suggesting that aBL is a very promising antimicrobial approach.

The photodynamic research community consists of various academic groups affiliated with two associations, the International Photodynamic Association (IPA) and the European Society of Photobiology (ESP), that are working to introduce aPDI and aBL in the clinic. Photodynamic research groups have made significant scientific progress, and their research on photodynamic inactivation has been published in desirable scientific journals, such as The Lancet (Wainwright et al., 2017), Science (Lu et al., 2021), and journals of the Nature Publishing Group (Wang et al., 2021). All of the findings indicate that aBL is a promising therapeutic alternative and may become a significant complementary treatment option to fight multidrug-resistant microbial infections. Despite the many advantages of aBL, this strategy is not frequently implemented in standard treatment procedures, possibly because the mechanism of action of aBL is not fully understood. The lack of a detailed understanding of the mechanism of action of aBL at the genetic level is a limitation and has hindered the widespread implementation of this effective method of bacterial eradication.

Transcriptomic research is one of the main branches of experimental research on the principle underlying the effect of aBL. Due to its high specificity, sensitivity and reproducibility, reverse transcription quantitative real-time polymerase chain reaction (RT‒qPCR) is a “gold standard” for gene expression quantification. However, the qPCR method depends on several variables that can affect the analysis process, such as the quantity and integrity of the extracted RNA, the sample amount and the designed primers. To overcome these variabilities, it is important to perform relative normalization, wherein the expression levels of target genes are normalized relative to the expression of a reference gene (selected as a stably expressed gene, most often a housekeeping gene). Therefore, the selection of an appropriate reference gene is crucial for relative gene expression studies (Guenin et al., 2009; Gomes et al., 2018). The use of different reference genes can produce surprisingly different gene expression results.

To date, there is little information on the selection of appropriate reference genes for the PDI and aBL process. To our knowledge, there are no published data on *Escherichia coli* gene stability after aBL treatment, and our research group has shown a great need to develop this as a basic method for gene expression research and to identify reference genes for the correct analysis and interpretation of the obtained results from aBL treatment. For this reason, the present study aimed to (1) determine the most stable reference gene under treatment with aBL under sublethal conditions, (2) evaluate differences in the expression of the selected gene after aBL treatment in comparison to that in untreated cells, and (3) compare differences in gene expression for two wavelengths of blue light, namely, 409 and 415 nm. In this study, 10 genes were selected as reference gene candidates (*arcA, cysG, gyrA, hcaT, idnT, ihfB, rpoA, rssA, uidA* and *uxuR*), encoding proteins involved in essential cellular processes. Additionally, 11 genes (*cpxA, deoB, dnaK, dnaJ, oxyR, pgi, purA, rbfA, umuD, ydcX,* and *yihE*), whose deletion resulted in hypersensitivity to aBL in single-gene Keio mutants, were chosen based on prior findings (Kruszewska-Naczk et al., 2024). Furthermore, 6 genes (*rfaC, rfaD, dacA, fabH, gmhB,* and *hldE*) potentially associated with resistance mechanisms were selected, and their expression was analyzed in wild-type bacteria.

## Materials and methods

### Bacterial strain and culture conditions

In this study, the *E. coli* BW25113 strain (*E. coli* Keio Knockout Parent Strain (Baba et al., 2006)) was investigated and cultured in Luria–Bertani (LB) broth (BTL, Łódź, Poland) or LB agar medium (A&A, Gdansk, Poland). Overnight cultures were prepared under aerobic conditions in an orbital incubator (Innova 40, Brunswick, Germany) at 150 rpm and cultured for 16–20 h at 37°C.

### Reagents

Primers were designed with the NCBI Pick Primers tool and ordered from the Institute of Biochemistry and Biophysics Polish Academy of Sciences (IBB PAN, Oligo.pl, Warsaw, Poland). The genes *arcA, cysG, gyrA, hcaT, idnT, ihfB, rpoA, rssA, uidA,* and *uxuR* were selected as reference gene candidates encoding proteins involved in basic cellular processes based on a literature search. The following genes were investigated for changes in expression levels under the influence of aBL: *cpxA, dacA, deoB, dnaK, dnaJ, fabH, gmhB, hldE, oxyR, pgi, purA, rbfA, rfaC (waaC), rfaD, umuD, ydcX (ortT),* and *yihE (srkA),* reported as aBL-hypersensitive genes in (Kruszewska-Naczk et al., 2024). All primer sequences are listed in Supplementary Table S1 in Supplementary Material, while the genes and their functions are listed in Table 1. Information about proteins encoded by genes was obtained from http://www.ecocyc.org.

**TABLE 1:** List of reference and investigated genes and their functions.

Reference genes			
Number	Gene	Encoded protein	Function
1	*arcA*	DNA-binding transcriptional dual regulator ArcA	regulation of DNA-templated transcription
2	*cysG*	siroheme synthase	response to osmotic stress, porphyrin-containing compound biosynthetic process
3	*gyrA*	DNA gyrase subunit A	DNA topological change
4	*hcaT*	putative 3-phenylpropionate transporter	lactose transport
5	*idnT*	L-idonate/5-ketogluconate/gluconate transporter	carbohydrate transport
6	*ihfB*	integration host factor subunit β	regulation of translation, DNA recombination, DNA-templated transcription
7	*rpoA*	RNA polymerase subunit α	DNA-templated transcription, response to heat, cellular response to cell envelope stress
8	*rssA*	putative patatin-like phospholipase RssA	lipid metabolic process
9	*uidA*	β-D-glucuronidase	glucuronoside catabolic process
10	*uxuR*	DNA-binding transcriptional repressor UxuR	DNA-templated transcription

Investigated genes			
Number	Gene	Encoded protein	Function
1	*cpxA*	sensor histidine kinase CpxA	response to radiation, cellular response to cell envelope stress, cell adhesion involved in biofilm formation, protein autophosphorylation
2	*dacA*	D-alanyl-D-alanine carboxypeptidase DacA	peptidoglycan metabolic process, regulation of cell shape, proteolysis
3	*deoB*	phosphopentomutase	DNA damage response
4	*dnaK*	chaperone protein DnaK	response to heat, cellular response to unfolded protein, DNA replication
5	*dnaJ*	chaperone protein DnaJ	DNA replication, protein folding and unfolding, response to heat
6	*fabH*	3-oxoacyl- [acyl carrier protein] synthase 3	fatty acid metabolic process
7	*gmhB*	D-glycero-β-D-manno-heptose-1,7-bisphosphate 7-phosphatase	lipopolysaccharide core region biosynthetic process
8	*hldE*	fused heptose 7-phosphate kinase/heptose 1-phosphate adenyltransferase	lipopolysaccharide core region biosynthetic process
9	*oxyR*	DNA-binding transcriptional dual regulator OxyR	DNA damage response, response to oxidative stress
10	*pgi*	glucose-6-phosphate isomerase	glycolytic process, cellular response to oxidative stress, gluconeogenesis
11	*purA*	adenylosuccinate synthetase	purine nucleotide biosynthetic process, DNA damage response
12	*rbfA*	30S ribosome binding factor	DNA damage response, ribosome biogenesis
13	*rfaC (waaC)*	ADP-heptose: LPS heptosyltransferase 1	lipopolysaccharide core region biosynthetic process
14	*rfaD*	ADP-L-glycero-D-mannoheptose 6-epimerase	lipopolysaccharide core region biosynthetic process
15	*umuD*	DNA polymerase V protein UmuD	DNA damage response, SOS response
16	*ydcX (ortT)*	orphan toxin OrtT	autolysis
17	*yihE (srkA)*	stress response kinase A	response to stress, phosphorylation

### Light sources

Two LED devices were used in the research. One had a λ_max_ of 415 nm and irradiance of 25 mW/cm^2^ (Cezos, Gdynia, Poland) (Kruszewska-Naczk et al., 2024), and the other had a λ_max_ of 409 nm and irradiance of 5.2 mW/cm^2^ (Cezos, Gdynia, Poland).

### aBL treatment and RNA isolation

**FIGURE 1:**
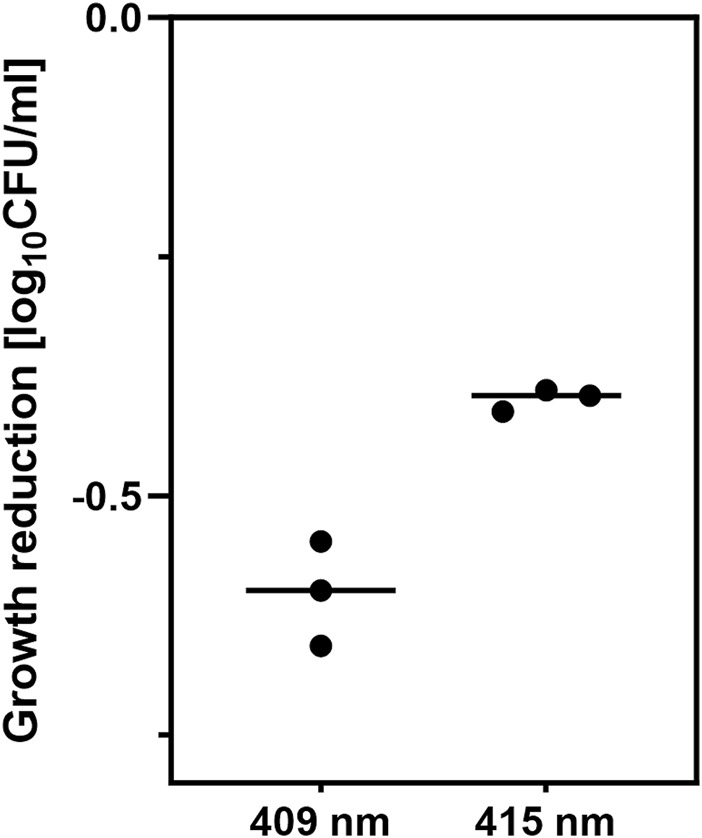
Growth reduction by aBL caused by 30 min of irradiation with two LED sources, 409 nm and 415 nm. The irradiation time was equal for both light sources. The experiments were performed in triplicate.

An overnight culture of the *E. coli* BW25113 strain was diluted 1:100 and incubated at 37°C and 150 RPM for 2 h to obtain an OD_600_ = 0.5. Next, 600 µL of the overnight culture was transferred to 24-well plates and irradiated for 30 min to reduce the bacterial load by ∼0.5 log_10_ CFU/mL. The dose of irradiation was chosen to achieve a similar reduction in the bacterial load and an equal duration of exposure to light. Thirty minutes of irradiation corresponds to 43.2 J/cm^2^ for the 415 nm LED and 9.36 J/cm^2^ for the 409 nm LED. Simultaneously, the control sample was incubated in the dark. Then, the samples were incubated for 40 min at 37°C in an orbital incubator (Innova 40, Brunswick, Germany) at 150 rpm. One set of controls used for reference gene selection was directly subjected to the next steps without resting. Next, 10 µL of each sample was diluted, streaked onto an LB agar plate, and incubated at 37°C for 16–20 h to count the surviving colonies. The results are shown in Figure 1. Five hundred microliters of the irradiated samples or nonirradiated controls were diluted in RNA protection reagent and incubated for 10 min, followed by centrifugation for 10 min at 5,000 × g. Bacterial pellets were frozen at −80°C. The experiments were performed in triplicate. RNA from bacterial pellets was isolated using the Syngen Blood/Cell RNA Mini Kit (Syngen, Poland). Genomic DNA contamination was removed using the TURBO DNA-free™ Kit (Thermo Fisher Scientific Baltics UAB, Lithuania). The quality of the isolated RNA was assessed by measuring its concentration and the 260/280 and 260/230 ratios using a NanoDrop spectrophotometer (Thermo Scientific, United States). A QuantiTect Reverse Transcription Kit (Qiagen, Germany) was used to transcribe RNA to cDNA. For each sample, 500 ng of RNA was collected to obtain the same amount of cDNA.

### qPCR-based gene expression analysis

Gene expression analysis was performed using a LightCycler 480 II (Roche Life Science, Germany). The optimal concentration of primers was selected based on qPCR Cp values. The optimal concentration for each pair of primers was 0.3 µM. The reaction mixture contained the following components: 5 µL of Fast SG qPCR Master Mix (EURx, Poland), 0.3 µM each primer pair (IBB Oligo.pl, Warsaw, Poland), nuclease-free water (EURx, Poland) and 1 µL of 25-fold diluted cDNA. To control contamination with genomic DNA, RNA was added to the reaction mixture instead of cDNA. In another reagent purity control, water was added instead of cDNA. The program consisted of the following steps: initial denaturation at 95°C for 5 min; 45 cycles of denaturation at 95°C for 15 s, annealing at 60°C for 15 s and elongation at 72°C for 15 s; final elongation at 72°C for 3 min; and melting curve generation at 65°C–95°C. To evaluate the specific amplification and possible dimer formation ability of the primers, melting curve analysis was performed. For the control samples not treated with aBL, standard curves were prepared for each primer (0 min of delay for reference gene candidates and 40 min of rest for investigated genes), and the reaction efficiency was calculated. For standard curves, five dilutions of cDNA (1:5, 1:25, 1:125, 1:625, 1:3125) prepared from the nonirradiated control were used. The following parameters were assessed and are listed in Supplementary Table S2 in Supplementary Material: efficiency and slope. The experiments were performed in four technical repetitions. As a reference gene *ihfB* was chosen. The PCR efficiencies for the investigated and reference genes were not equal, so the Pfaffl gene expression analysis model was implemented according to the following equation:

$$R=\frac{{\left(E_{target\;gene}\right)}^{∆Cp\;target\;gene\;\left(control-sample\right)}}{{\left(E_{reference\;gene}\right)}^{∆Cp\;reference\;gene\;\left(control-sample\right)}}$$

where R is the gene expression ratio, E_target_ is the reaction efficiency for the investigated gene, E_reference gene_ is the reaction efficiency for the reference gene, ∆Cp is the difference between the Cp values for the control and irradiated samples for the investigated gene, and ∆Cp is the difference between the Cp values for the control sample and the irradiated reference gene sample (Pfaffl, 2001). The calibrator control was a nonirradiated sample, which was normalized to 1. All R values were log_2_ transformed and plotted as a fold change in expression level, where a value of 0 represents the expression level of the control. The R value was calculated for 3 independent biological replicates with standard deviation.

### Statistical analysis and graphs

The calculations were performed in Excel, while the figures and statistical analysis were performed with GraphPad Prism version 9.0 (GraphPad Software, Inc., CA, United States). The significant differences between the groups were calculated using two-way analysis of variance (ANOVA) with P < 0.05 and Dunnett multiple comparison tests. The figures were prepared with BioRender.com (accessed on 27 May 2024).

### Selection of stable reference genes

This step was performed using 4 programs: geNorm (Vandesompele et al., 2002), BestKeeper (Pfaffl et al., 2004), NormFinder (Andersen et al., 2004), and RefFinder (Xie et al., 2012; Xie et al., 2023) with the comparative delta-Ct method (Silver et al., 2006). The obtained Cp values were used for the analysis performed with ReFinder and BestKeeper. For the analysis with geNorm and NormFinder, the Cp values were normalized according to the Cp of the control samples and the reaction efficiency for each primer pair. Four conditions for WT strain treatment were implemented in three biological and four technical repetitions: 0 and 40 min of resting without aBL treatment (control) and 40 min of resting after aBL 409 nm and aBL 415 nm treatment.

## Results

**FIGURE 2:**
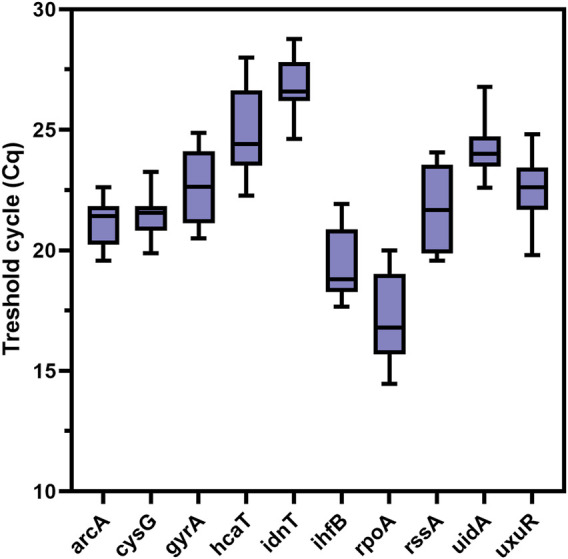
Distribution of reference gene data for threshold cycles.

The results of the ReFinder reference gene analysis are presented in Table 2, the BestKeeper analysis in Table 3, and the geNorm and NormFinder analyses in Table 4. In Figure 2 distribution of reference gene data for threshold cycles was presented. The threshold cycle values for the most stable reference gene candidates were close to 20 for the best candidates (Figure 2).

**TABLE 2:** ReFinder analysis results.

Gene	Comprehensive ranking (Geomean ranking values)	Delta Ct method (average of standard deviation)	Bestkeeper Cp (standard deviation)	NormFinder (stability value)	geNorm (stability value)
*arcA*	4.56	1.56	0.78	1.001	1.235
*cysG*	2.45	1.4	0.667	0.708	1.098
*gyrA*	4.86	1.48	1.19	0.847	1.182
*hcaT*	8	1.72	1.49	1.345	1.348
*idnT*	3.16	1.49	1	0.954	0.793
*ihfB*	1.57	1.31	1.14	0.625	0.793
*rpoA*	9	1.89	1.51	1.579	1.435
*rssA*	7.65	1.68	1.67	1.23	1.287
*uidA*	2.45	1.4	0.83	0.692	0.969
*uxuR*	8.41	2.45	1.06	2.257	1.639

**TABLE 3:** BestKeeper analysis results. Bold values meet the criteria set by the program.

	*arcA*	*cysG*	*gyrA*	*hcaT*	*idnT*	*ihfB*	*rpoA*	*rssA*	*uidA*	*uxuR*
n	12	12	12	12	12	12	12	12	12	12
geo Mean [CP]	21.19	21.51	22.65	24.90	26.68	19.29	17.07	21.68	24.17	22.47
ar Mean [CP]	21.21	21.53	22.70	24.96	26.71	19.34	17.15	21.75	24.19	22.51
min [CP]	19.57	19.89	20.50	22.28	24.63	17.66	14.47	19.58	22.61	19.81
max [CP]	22.62	23.26	24.87	27.99	28.77	21.93	20.00	24.07	26.78	24.82
std dev [±CP]	**0.80**	**0.69**	1.28	1.57	**0.95**	1.14	1.39	1.59	**0.80**	1.03
CV [% CP]	3.77	3.21	5.65	6.28	3.55	5.89	8.10	7.31	3.30	4.57
min [x-fold]	−3.04	−2.93	−3.78	−5.28	−4.30	−3.09	−5.78	−4.00	−3.23	−5.77
max [x-fold]	2.67	3.19	3.94	7.13	4.40	6.24	7.20	4.84	7.13	4.75
std dev [±x-fold]	**1.73**	**1.60**	2.41	2.93	**1.91**	2.18	2.59	2.97	**1.73**	2.02
coeff. of corr. [r]	0.526	**0.723**	0.82	0.712	0.746	**0.955**	0.665	**0.878**	0.673	−0.506
p value	0.078	0.008	0.001	0.009	0.005	0.001	0.018	0.001	0.016	0.092

**TABLE 4:** Results of the geNorm and NormFinder reference gene analyses. Bold values meet the criteria set by the program.

Gene	geNorm - M value	NormFinder - stability value
*arcA*	**1.446**	0.232
*cysG*	**1,312**	0.211
*gyrA*	**1.410**	0.190
*hcaT*	1.648	0.232
*idnT*	**1.403**	0.252
*ihfB*	**1.258**	**0.158**
*rpoA*	1.755	0.200
*rssA*	1.506	0.262
*uidA*	**1.421**	0.216
*uxuR*	2.333	0.310
*cysG-gyrA*	-	0.127

### *ihfB* is the gene with the most stable expression after aBL treatment

**FIGURE 3:**
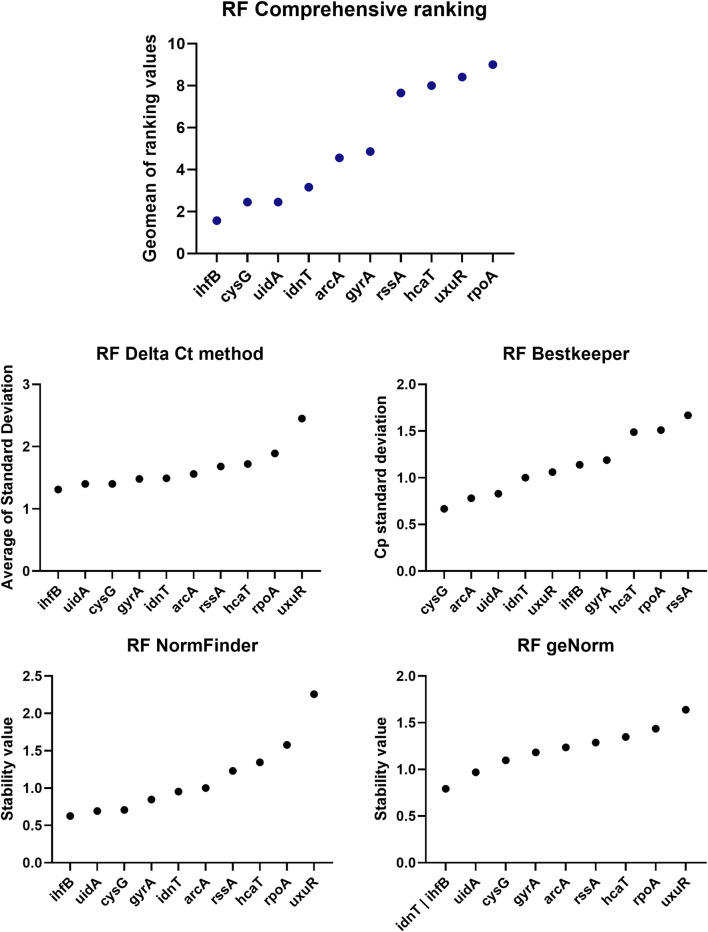
ReFinder (RF) analysis results. The comprehensive ranking consolidates all results into a single plot, utilizing the methodologies implemented in the ReFinder program.

The *ihfB* gene encoding the integration host factor β subunit was confirmed to be the best reference gene candidate for aBL treatment at both 409 and 415 nm. Three programs, RefFinder, geNorm, and NormFinder, indicated that this gene had the most stable expression in comparison to the other reference candidates. The next best candidates were *cysG, uidA* and *gyrA* with parameters similar to *ihfB* (Table 2). NormFinder revealed *ihfB* as the single gene and *cysG*-*gyrA* as the combination of reference genes with the greatest stability. The results from geNorm and NormFinder with data normalized according to the reaction efficiency and gene expression in the control were not comparable to the results of the algorithms used in RefFinder. However, in the final ranking, both approaches revealed the same genes as the best and worst candidates. ReFinder and BestKeeper were used to analyze the Cp values without normalization. BestKeeper was used to determine the reaction efficiency for analysis. According to the BestKeeper criteria, the standard deviation of Cp should be less than 1, the standard deviation of the regulation coefficient should be less than 2, and the correlation coefficient should be close to 1 for *arcA, cysG, idnT,* and *uidA,* with the best values observed for *cysG* (Table 3). According to geNorm and its automated settings, the most stable genes with M values less than 1.5 were arcA*, cysG, gyrA, hcaT, idnT, ihfB*, and uidA*,* while *ihfB* had the lowest value. M values above 1.5 was suggested as indicating the least stable candidates. NormFinder analysis revealed that only the *ihfB* gene met all the program criteria (Table 4). According to the comprehensive ranking and the delta Ct results calculated by RefFinder, *ihfB* was also the best candidate (Figure 3; Table 2).

### There were no statistically significant differences in gene expression between the aBL 409 and 415 nm treatments for most of the investigated genes

**FIGURE 4:**
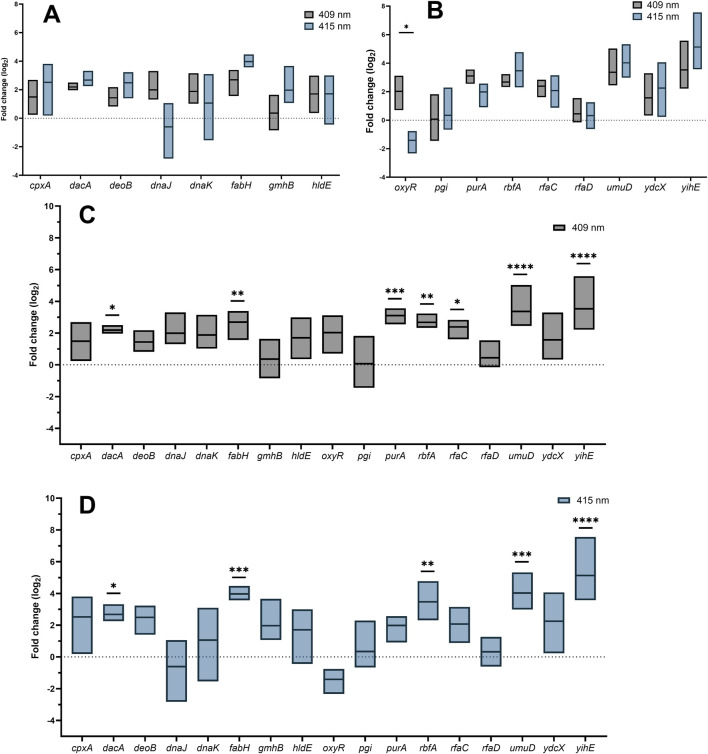
**(A, B)** Comparison of gene expression between *E. coli* treated with 409 nm and 415 nm aBL. Fold change in the expression of investigated genes after treatment with 409 nm **(C)** and 415 nm **(D)** aBL. P values indicating statistical significance are indicated with asterisks (ns P > 0.05; *P < 0.05; **P < 0.01; ***P < 0.001; ****P ≤ 0.0001).

The expression levels of aBL-hypersensitive genes after aBL treatment at both wavelengths were statistically similar. A difference was noted only for the *oxyR* expression level. After aBL 409 nm irradiation, *oxyR* expression was significantly greater than that after aBL 415 nm treatment (Figures 4A, B).

### The expression of *dacA, fabH, rbfA, umuD, and yihE* increased after aBL treatment for both wavelengths of blue light

The patterns of gene expression changes after aBL irradiation at both wavelengths were similar. The expression levels of five of the seventeen genes, *dacA, fabH, rbfA, umuD,* and *yihE*, were significantly greater than those in the control. The expression levels of *purA* and *rfaC* increased only after treatment with aBL 409 nm. The greatest differences were noted for *fabH, purA, rbfA, umuD* and *yihE*. The remaining genes investigated, *cpxA, deoB, dnaJ, dnaK, gmhB, hldE, oxyR, pgi, rfaD, and ydcX,* were not expressed at significantly higher levels (Figures 4C, D).

## Discussion

Ogonowska et al. (2020) reported that there is no information about the selection of suitable reference genes under aPDI. In the study, the authors provided a list of candidate reference genes stably expressed under aPDI mediated with two different photosensitizers. First, they selected *gmk* and *ftsZ* for rose bengal-mediated aPDI with the use of green light and *ftsZ, proC*, and *fabD* for new methylene blue-mediated aPDI with the use of red light. Next, the research group used the selected stable reference gene candidates for measuring the expression levels of the *Staphylococcus aureus* enterotoxin b gene (*seb*) under two photodynamic treatment conditions (Ogonowska and Nakonieczna, 2020).

Because there are no published data on *E. coli* genes that are stable under aBL treatment, the present study aimed to identify a stable reference gene after aBL treatment at two light wavelengths, 409 and 415 nm, to investigate the role of the gene in antimicrobial mechanisms. To our knowledge, no such candidate stable gene has been reported to date. We found several studies evaluating stable genes whose expression does not vary under various stress conditions in *E. coli* and investigated whether these genes are also stable under blue light stress, which has a multitarget mode of action. Peng et al. (2014) evaluated the genes *hcaT* (encoding a uroporphyrin III C-methyltransferase), *cysG* (encoding a 3-phenylpropionic transporter) and *rssA* (encoding a 16S ribosomal RNA) as reference gene candidates whose expression is stable under salt and organic acid stress. RNA was isolated from bacteria in the stationary phase of growth, and the results were analyzed using the BestKeeper tool. The most stable gene was *rssA, and* the least stable gene was *cysG* (Peng et al., 2014). In our study, the stability tendency after aBL treatment was the opposite, where *cysG* was a better candidate reference gene than *rssA* and *hcaT*. In another study, Zhou et al. (2011) identified novel reference genes for testing gene transcription in recombinant protein-producing *E. coli.* Two bioinformatics tools were used: geNorm and NormFinder. The researchers tested the expression of twenty genes at 28°C and 37°C under different induction conditions. The analysis revealed three genes as the most reliable: *cysG, hcaT* and *idnT* (encoding the L-idonate/5-ketogluconate/gluconate transporter). Moreover, the *rssA* and *ihfB* genes (encoding the integration host factor subunit β) were unstable under different growth temperatures and induction conditions. The following results also do not correspond to our studies on aBL (Zhou et al., 2011). Studies by Teixeira et al. (2016) investigated stable gene expression after laser irradiation (660 and 808 nm) at different fluences. Gene expression of *E. coli genes* in the exponential growth phase was studied. The BestKeeper, geNorm and NormFinder programs were used for stability analysis. When comparing the cycle-threshold values, no significant difference was noted in the expression of the reference genes for the irradiated sample and controls. However, Excel-based tools did not demonstrate the stability of *arcA, gyrA,* or *rpoA* expression after red and infrared laser treatment. The authors suggested that the investigated conditions modulated the mRNA levels of the tested reference genes (Teixeira et al., 2016). Our analysis revealed that *arcA, gyrA, and rpoA* are also not the best reference gene candidates for aBL treatment. In another study conducted by Walker et al. (2017), three genes, namely, *ybbW* (encoding allantoin transporter), *uidA* (encoding β-D-glucuronidase) and *clpB* (encoding chaperone protein ClpB), were considered targets for detecting *E. coli* contamination in water. In the tested isolates, *ybbW* was detected by qPCR, while other investigated genes were not detected in any of the tested isolates (Walker et al., 2022).

In our previous studies, we screened for aBL sensitivity among *E. coli* mutants lacking nonessential genes. Among them, we identified 64 aBL-hypersensitive genes important in the bacterial response to blue light (Kruszewska-Naczk et al., 2024). In this study, 17 of the 64 aBL-hypersensitive genes were selected for gene expression analysis (*cpxA, deoB, dnaK, dnaJ, oxyR, pgi, purA, rbfA, umuD, ydcX,* and *yihE*, whose deletion resulted in hypersensitivity to aBL in single-gene Keio mutants and 6 additional genes (*rfaC, rfaD, dacA, fabH, gmhB,* and *hldE* that could potentially contribute to the development of resistance). Previous studies utilized mutants complemented with the deleted genes (11 complementation mutants with restored gene function). Complementation not only restored wild-type sensitivity to aBL but, in some cases, rendered mutants less sensitive to aBL than the wild-type strain (Kruszewska-Naczk et al., 2024).

These included genes from several categories depending on the function of the encoded protein: response to DNA damage, oxidative stress response, lipopolysaccharide production, fatty acid metabolic process, response to cell envelope stress, autolysis, radiation, heat, and SOS response. Four investigated genes were involved in the biosynthesis of the lipopolysaccharide core region (*gmhB, hldE, rfaC, and rfaD*), and only one of them, *rfaC,* was significantly highly expressed. Not all investigated genes responsible for the response to DNA damage were characterized by significantly increased expression. The expression levels of *purA, rbfA* and *umuD* were greater than those of *deoB* and *oxyR*. This may suggest that not all repair systems are engaged in cell protection against aBL at sublethal doses of irradiation. The expression pattern may differ under higher light doses, as bacteria employ more strategies to survive light stress.

Stress generation can be observed through the expression of *yihE*, which encodes stress response kinase A (SrkA). This kinase exhibits autophosphorylation activity at serine and threonine residues (Zheng et al., 2007). SrkA is also regulated by the CpxR system, which is a part of the cellular response to many types of environmental stresses that lead to bacterial adaptation (Zhao et al., 2022). Additionally, *fabH* was expressed at significantly higher levels. This gene plays a significant role in the process of fatty acid biosynthesis. This is one of the targets of novel antimicrobial agents, including carbazole derivatives, dioxygenated amide derivatives, and Platencin (Chen et al., 2024; Chang et al., 2024; Wang et al., 2007).

Our previous study results (Kruszewska-Naczk et al., 2024) suggested that cell protection against aBL is an active process, which means that it has an energy cost for bacterial cells. During aBL irradiation, bacteria can actively produce ATP and use the energy reservoir fatty acids, which leads to the activation of genes that synthesize the depleted fatty acids (Olukoshi and Packter, 1994).

Fatty acids can also act as signal molecules that may be involved in the activation of cellar protective pathways. Moreover, fatty acids are a component of bacterial membranes. Chu et al. (2019) revealed that aBL causes damage to fatty acids via oxidation. Changes in cellular fatty acid profiles were also confirmed, along with a decrease in the amount of unsaturated fatty acids (Chu et al., 2019). Wu et al. (2018) demonstrated changes in membrane permeability after aBL treatment. Research conducted by dos Anjos et al. (2023) indicated changes in bacterial shape after aBL treatment, suggesting membrane damage.

Another highly expressed gene was *umuD*, which is involved in the bacterial SOS response. The UmuD′2C protein complex is an error-prone DNA polymerase, and RecA interacts with the DNA polymerase III holoenzyme, which repairs DNA damage. UmuD′2C has been reported to act as a DNA damage checkpoint system and regulate the appropriate timing and level of DNA synthesis in bacteria under DNA damage stress (Frank et al., 1993; Sutton et al., 1999). This may also confirm that aBL causes DNA damage and activation of repair systems. For instance, dos Anjos et al. (2023) observed DNA degradation upon aBL treatment.

*dacA* was another highly expressed gene after aBL irradiation. DacA, i.e., penicillin-binding protein 5 (PBP5), is involved in the synthesis of peptidoglycan and is also responsible for cell shape (Nelson and Young, 2000). Additionally, deletion of this gene sensitizes bacteria to penicillin (Sarkar et al., 2010). The results presented by Choi et al. (2023) indicated that DacA plays a crucial role in growth under salt stress and a secondary role in bacterial growth under alkaline stress conditions. *dacA* may play a significant role in bacterial protection against aBL because aBL causes cell wall deformations that can be repaired by DacA (Zhang et al., 2016).

Contrary to the findings of Walker et al. (2022), we did not observe increased expression of *dnaK*, which is involved in the heat stress response. Previous studies reported the upregulation of the *dnaK* gene in *Campylobacter jejuni* treated with aBL at 405 nm (Walker et al., 2017).

An equal irradiation time for both wavelengths was used to compare changes in gene expression so that bacteria had the same amount of time to activate the expression of protective genes. The survival efficiency of bacteria after exposure was also similar. However, the light sources differed in terms of irradiance. The irradiance of the 409 nm LED was 5.2 mW/cm^2^, while that of the 415 nm LED was 25 mW/cm^2^. Compared with those of the control, significantly greater levels of gene expression were detected for the light source with a wavelength of 409 nm and lower irradiance. The difference in the gene expression pattern between the two wavelengths may be due to a shift in the absorption peak of endogenous porphyrins, which results in the activation of additional cell protection mechanisms, resulting in the expression of more genes at 409 nm aBL (Figures 4A‐D). Notably, statistical comparison of expression levels between the two light sources revealed differences only for *oxyR* (Figures 4A‐D). The expression level of this gene was greater for 409 nm aBL than for 415 nm aBL. The OxyR regulon controls the expression of genes involved in the oxidative stress response (Tartaglia et al., 1989). One hypothesis to explain why the expression level of *oxyR* may be greater for 409 nm aBL is that it likely generates more ROS than 415 nm aBL. However, this should be checked using a special detection probe.

In summary, there are no universal reference genes whose expression does not vary and is applicable under every stress condition. Therefore, there is a need to evaluate reference genes for every tested stressor. For aBL at different wavelengths, we found universal genes with stable expression after irradiation.

## Data Availability

The data presented in the study are deposited in the Zenodo repository: https://zenodo.org/records/14506916.
