# Supplementary material for: Identification and validation of reference genes for quantitative gene expression analysis under 409 and 415 nm antimicrobial blue light treatment
Source: Front Mol Biosci. 2025 Jan 6;11:1467726. doi: 10.3389/fmolb.2024.1467726 (PMC11743365; doi:10.3389/fmolb.2024.1467726)
Supplement: Supplementary file 1 [file Table1.docx]

**Table S1. List of primers used in the study.**

| **Reference genes** | | | |
| --- | --- | --- | --- |
| **Number** | **Gene** | **Forward primer** | **Reverse primer** |
| 1. | *arcA* | AACGGCTGTTGATGTCCAGT | GCAACCTACTGTCCCGTACC |
| 2. | *cysG* | TTGTCGGCGGTGGTGATGTC | ATGCGGTGAACTGTGGAATAAACG |
| 3. | *gyrA* | ACGCGACTTGGTTGGGTATT | GTCTCTCTGATCGTGCCTCG |
| 4. | *hcaT* | GCTGCTCGGCTTTCTCATCC | CCAACCACGCTGACCAACC |
| 5. | *idnT* | CTGTTTAGCGAAGAGGAGATGC | ACAAACGGCGGCGATAGC |
| 6. | *ihfB* | CACCCAGCAATCGCACATTC | GTCGAGGCCATATGCTCCAG |
| 7. | *rpoA* | ACGACGAATCGCCTCTTCAG | CGTGTAGAACAGCGTACCGA |
| 8. | *rssA* | GATGGATCTCTCCTGGCAGC | TCCGGCATTATTTCGCGGTA |
| 9. | *uidA* | TTGTCCAGTTGCAACCACCT | AGACTGTAACCACGCGTCTG |
| 10. | *uxuR* | AAATGCTTGATGTCACGCGG | CGTACTTCCACCAGCCCTTT |
| **Investigated genes** | | | |
| **Number** | **Gene name** | **Forward Primer** | **Reverse Primer** |
| 1. | *cpxA* | CCAAACCTGTACCGCCAGAT | CGCGAACAGATTTTCCGTCC |
| 2. | *dacA* | CGGTTCAGAGGCGAACTCTT | CTAACCTGGGGCTTCCGTTT |
| 3. | *deoB* | CTGCCAAATCTGACCCGTCT | CGATAACTTCAGCGTTGCCG |
| 4. | *dnaK* | ATGACCTGGGTGGTGGTACT | CGTTGGTTGCCAGAACTTCG |
| 5. | *dnaJ* | GCCGCTAAGCAAGATTATTACGA | CCGCGGGTCAGGTCGTCAAAAAA |
| 6. | *fabH* | GCGCACATCGTTGATGAGAC | AGGTTAGCCTGATGCGGAAC |
| 7. | *gmhB* | CATCCGCAGGGTAGTGTTGA | CGTGCTGACAAAAGCATCCC |
| 8. | *hldE* | TTGACGGCAACAATCAAGCG | GGTGATGACCAACGGTGTCT |
| 9. | *oxyR* | GCCAATATTCGTGATCTTGAGTA | CCAACCGCCTGTTTTAAAACTTT |
| 10. | *pgi* | AGCTCTGCGTCCGTACAAAA | CAAGAACAGCGTGGTTTCCG |
| 11. | *purA* | ACAACGCAGGCCATACTCTC | ACCGTTACCGATGATGCTGG |
| 12. | *rbfA* | CGTCATGTTTGACCACGCTG | TGCCGGAACTGACCTTCTTC |
| 13. | *rfaC (waaC)* | TGGGCGATGTTCTCCATACG | TCTGTGCGAACCCTTCTTCC |
| 14. | *rfaD* | GCGTCGCTTTCCATCTCAAC | CCACATAGACGAAGTCGCGT |
| 15. | *umuD* | GCCCGACGGTACAGCTTATT | ACACCAAAGACATCCAGCGT |
| 16. | *ydcX (ortT)* | TGCGGTTATGGCAGCAATCG | ATGTTGCCCCCACCAGAAAG |
| 17. | *yihE (srkA)* | TATTCTCTGGCGCGATGGTC | GCTCGGCTTTATCGCCATTG |

**Table S2. Parameters for validation of the qPCR for each gene.**

| **Reference genes** | | | | |
| --- | --- | --- | --- | --- |
| **Number** | **Gene** | **Slope** | **Efficiency** | **Efficiency [%]** |
| 1. | *arcA* | -3,361 | 1,984 | 99,2 |
| 2. | *cysG* | -3,478 | 1,939 | 96,95 |
| 3. | *gyrA* | -3,725 | 1,856 | 92,8 |
| 4. | *hcaT* | -3,619 | 1,889 | 94,45 |
| 5. | *idnT* | -3,24 | 2,035 | 101,75 |
| 6. | *ihfB* | -3,325 | 1,999 | 99,95 |
| 7. | *rpoA* | -3,415 | 1,962 | 98,1 |
| 8. | *rssA* | -3,485 | 1,936 | 96,8 |
| 9. | *uidA* | -3,061 | 2,122 | 106,1 |
| 10. | *uxuR* | 3,48 | 1,936 | 96,8 |
| **Investigated genes** | | | | |
| **Number** | **Gene** | **Slope** | **Efficiency** | **Efficiency [%]** |
| 1. | *cpxA* | -3,492 | 1,934 | 96,7 |
| 2. | *dacA* | -3,292 | 2,013 | 100,65 |
| 3. | *deoB* | -3,014 | 2,147 | 107,35 |
| 4. | *dnaK* | -3,397 | 1,97 | 98,5 |
| 5. | *dnaJ* | -3,918 | 1,8 | 90 |
| 6. | *fabH* | -3,58 | 1,903 | 95,15 |
| 7. | *gmhB* | -3,336 | 1,994 | 99,7 |
| 8. | *hldE* | -3,157 | 2,074 | 103,7 |
| 9. | *oxyR* | -3,901 | 1,804 | 90,2 |
| 10. | *pgi* | -3,461 | 1,945 | 97,25 |
| 11. | *purA* | -3,352 | 1,987 | 99,35 |
| 12. | *rbfA* | -3,369 | 1,981 | 99,05 |
| 13. | *rfaC (waaC)* | -3,498 | 1,983 | 0 |
| 14. | *rfaD* | -3,498 | 1,932 | 96,6 |
| 15. | *umuD* | -3,502 | 1,93 | 96,5 |
| 16. | *ydcX (ortT)* | -3,352 | 1,987 | 99,35 |
| 17. | *yihE (srkA)* | -3,438 | 1,954 | 97,7 |
